# Characterization of Prejunctional Muscarinic Receptors: Effects on the Release of VIP and Functional Responses and Receptor Expression in the Ovine Submandibular Gland

**DOI:** 10.1155/2009/787586

**Published:** 2009-05-25

**Authors:** Anders T. Ryberg, Ondrej Soukup, Gunnar Tobin

**Affiliations:** ^1^Department of Pharmacology, Institute of Neuroscience and Physiology, The Sahlgrenska Academy, University of Gothenburg, 405 30 Göteborg, Sweden; ^2^Department of Toxicology, Faculty of Military Health Sciences, University of Defence, 500 01 Hradec Kralove, Czech Republic

## Abstract

In the in vivo experiments on anaesthetized sheep, it was presently examined whether muscarinic receptor antagonists with diverse selectivity affect the release of VIP in response to electrical stimulation of the parasympathetic chorda tympanic nerve differently, and if the changes in the release could be associated to altered secretory and vasodilator responses. The location of the muscarinic receptor subtypes was examined also. In the experiments, blood was collected out of the submandibular venous drainage before and during electrical stimulation of chorda tympani nerve in the absence and presence either of pirenzepine or methoctramine. While metchoctramine increased the output of protein, pirenzepine inhibited flow of saliva and increased protein output, vasodilatation, and VIP output. In morphological examinations, the inhibitory muscarinic M4 receptor occurred interacinarily in the gland. It is concluded that prejunctional muscarinic receptors, most likely of the M4 subtype, exert inhibitory modulation of the parasympathetic release of VIP in the ovine submandibular gland.

## 1. Introduction

The fluid secretory response of the ovine submandibular gland to acetylcholine is exerted via both muscarinic M1 and M3 receptors, while M5 receptors also seem to participate in the cholinergic vasodilator response [1]. However, in the parasympathetic glandular neurons, the neuropeptide vasoactive intestinal peptide (VIP) may be colocalised with acetylcholine [2]. In the submandibular gland of the sheep, VIP is present in nerve terminals adjacent to both small blood vessels and acini [3]. In this gland as well as in the ovine parotid gland, VIP mediates secretion of protein-rich submandibular saliva, in addition, the vasodilator effects [4–6]. However, VIP is remarkably potent in eliciting protein secretion from salivary glands in a number of species [7–11], and VIP seems to play a role in human submandibular glands also, both regarding secretion and vasodilatation [12–14]. At the postjunctional level, VIP and acetylcholine interact and at simultaneous administration of the exogenous VIP and a muscarinic agonist, conspicuous positive synergies emerge [7, 8, 15]. The crosstalk between the two transmitter substances occurs at prejunctional level also [6, 16, 17]. In the ovine submandibular gland, intravenous injections of the “M2/4”-selective antagonist methoctramine significantly increased the parasympathetic nerve-evoked secretion of protein [1]. Also, unselective muscarinic receptor blockade of prejunctional receptors has been shown in a number of species to increase VIPergic responses together with the release of VIP upon electrical stimulation of the parasympathetic glandular innervation [6, 18, 19]. The effect of the blockade of the prejunctional receptors seems to be unspecific and affects both the release of the neuropeptide VIP and the classical parasympathetic transmitter acetylcholine [17]. In contrast to acetylcholine, VIP is preferentially released during intense parasympathetic stimulation [20]. Therefore it was presently wondered whether or not blockade with muscarinic receptor antagonists with different selectivity profile affects the release of VIP in response to electrical stimulation of the parasympathetic chorda tympanic nerve differently, and, if so, the changes in the release could be associated to alterations in secretory and vasodilator responses. For this purpose blood was collected out of the submandibular venous drainage before and during electrical stimulation of the ovine chorda tympani nerve at a high frequency (8 Hz) in the absence and presence either of the muscarinic “M1-selective” receptor antagonist pirenzepine or the muscarinic “M2/4-selective” receptor antagonist methoctramine [21]. In order to look for morphological correlates to the functional findings, the expression and cellular location of the different muscarinic receptors were assessed by immunohistochemistry.

## 2. Methods

### 2.1. Animals

The experiments were carried out on 11 adult ewes of various breeds (35–72 kg body weight) under the Animals Scientific Procedures Act (1986); Project Licence PPL 80/1316. The in vivo experiments were performed at the Physiological Laboratory, Cambridge University, and the ethics Committee of the Cambridge University approved the study design. Food but not water was withheld for 48 hour prior to each experiment. Anaesthesia was induced and maintained with sodium pentobarbitone (Sagatal, Rhône Mérieux Ltd., Harlow, UK; 15–30 mg kg^−1^ IV (via a catheter in the femoral vein) and then 0.1–0.3 mg min^−1^ kg^−1^ IV (adjusted to maintain a stable blood pressure)). Surgery was performed as been described previously [1]. In short, the trachea was intubated, and the ipsilateral ascending cervical sympathetic nerve was cut. Via a catheter in the femoral artery, the arterial blood pressure was monitored. The chorda-lingual nerve was exposed and cut, and the submandibular duct was cannulated. No spontaneous flow of saliva occurred. Each of the tributaries of the ipsilateral linguofacial vein, except that draining the submandibular gland, was ligated. The animal was heparinized (Mutiparin, CP Pharmaceuticals, Wrexham, UK; 1000 IU kg^−1^ IV), the linguofacial vein cannulated, and the submandibular venous effluent diverted through a photoelectric drop counter and returned to the animal by a pump. Finally, a bipolar platinum stimulating electrode was placed under the duct and the chorda tympani close to the hilum of the gland. The protocol involved parasympathetic stimulation at 8 Hz continuously for 10 minutes (20-V square wave; 10-ms pulse width). At the end of each experiment the animal was given a lethal dose of barbiturate (Pentoject, Animalcare Ltd., York, UK; *ca* 15 mL 20% w/v), and the contra-lateral submandibular gland dissected out and weighed (12 ± 1 g; *n* = 11). Regarding the samples of blood, these were weighed for gravimetric estimation of blood flow and then returned to the animal to preserve the circulating blood volume, except for that volume of submandibular venous effluent blood kept for VIP estimations. The gravimetrical measurement ensured a high degree of accuracy since the blood flow occasionally increased so much that the drop counter did not discriminate between the single drops; all blood flow data presented are calculated from the gravimetrical measurements. Arterial blood samples were collected from the femoral artery at intervals for calculations of the glandular release of VIP into the circulation; the difference between arterial and venous VIP concentration, which is the actual data presented in the result section. The samples were collected into chilled preweighed tubes containing aprotinin (2500 KIU mL blood^−1^) and then centrifuged at +4°C as soon as possible and the plasma sequestered at −20°C. Plasma VIP concentrations were measured by an enzyme immunoassay (EIA for VIP, Peninsula Laboratories Inc., Calif, USA). The minimum detectable concentration for VIP was 0.02 pmol mL^−1^ (range 0–7.6 pmol mL^−1^; linear range 0.03–0.61 pmol mL^−1^). The saliva samples were analyzed for its protein content by the Lowry method [22]. Regarding the protein secretion, this is given as the protein output and thus disregarding the salivary flow rate; that is, it is not given as protein concentration.

### 2.2. Immunohistochemistry

After administration of a lethal dose of anesthetic, tissue from the contralateral submandibular gland was dissected out from the animal for histological examinations. A part (central, lower part) of glandular tissue of the parts most proximal to the glandular hilus was removed. The specimens were fixed in phosphate buffered 4% paraformaldehyde (pH 7.0), and then embedded in paraffin. 

For the immunohistochemical investigation of muscarinic receptor expression, transverse sections of the different specimens were prepared in a cryostat at a thickness of 4 *μ*m. The sections were deparaffinized by heating the slides to 60°C for 15 minutes and then subjected to two 30-minute changes in 100% xylene; the sections were then rehydrated by serial incubations in 100%, 95%, 85%, and 70% ethanol, followed by tris-buffered saline (TBS). Then the sections were immersed in 10 mM citrate buffer (pH 6.0) and were microwaved for four cycles of 4 minutes. Endogenous peroxidase was blocked with 0.03% hydrogen peroxidase for 30 minutes. Nonspecific protein binding was blocked with 5% bovine serum albumin (BSA) in TBS for 30 minutes. The sections were thereupon incubated overnight at 4°C in a humidified chamber with polyclonal rabbit anti-mAChR subtype specific antibodies (Research and Diagnostic Antibodies, Berkley, USA) diluted 100x in TBS containing 1% BSA. The presence of the muscarinic receptors was revealed using an avidin-biotin-complex immunoperoxidase method (ABC Staining System, Santa Cruz Biotechnology, Santa Cruz, USA; system used following the manufacturer's instructions) that uses 3,3P-diaminobenzidine (DAB) as a substrate. The sections were counterstained using Mayer's hematoxylin. As a negative control, duplicate sections were immunostained without exposure to the primary antibody, which resulted in no brown staining of the tissue.

### 2.3. Estimations

Submandibular vascular resistance (SVR) was estimated by dividing the perfusion (arterial blood) pressure (mmHg) by the submandibular blood flow (*μ*l min^−1^ [g gland]^−1^) and expressed as the % changes. Results are expressed as mean values ± S.E.M. and were assessed statistically by means of two-way ANOVA followed by posttest of Bonferroni. All flows and outputs are expressed per unit weight of the contralateral gland.

## 3. Results

### 3.1. Vasodilator and Secretory Responses to Stimulation of the Parasympathetic Innervation

In the absence of stimulation, the submandibular gland was quiescent according to secretion, while the mean basal glandular blood flow was 0.26 ± 0.04 mL min^−1^g gland^−1^ in the group (*n* = 6) in which the effect of pirenzepine was examined. In the group examining the effect of methoctramine the basal blood flow was 0.32 ± 0.05 mL min^−1^g gland^−1^ (*n* = 5). Also, the blood pressure was almost identical in the two groups (93 ± 5 vs 91 ± 4 mmHg), and neither antagonist affected the pressure. Electrical stimulation of the chorda tympani at 8 Hz evoked a mean flow of saliva over the 10-minutes stimulation at of 78 ± 3 and 59 ± 4 *μ*l min^−1^ g gland^−1^ before administration of pirenzepine and methoctramine, respectively. Correspondingly, the mean decreases in submandibular vascular resistance in the absence of antagonist were −78 ± 0.5 and −72 ± 0.5%, respectively. These changes in vascular resistance reflected mean blood flow during the stimulation of 1.23 ± 0.06 (in absence of antagonist; pirenzepine group) and 1.19 ± 0.05 mL min^−1^g gland^−1^ (in absence of antagonist; methoctramine group). The mean protein output over the stimulation period was in the two groups 61 ± 9 and 58 ± 7 *μ*g min^−1^ g gland^−1^, respectively.

### 3.2. Responses Following the Administration of Muscarinic Antagonists

**Figure 1:**
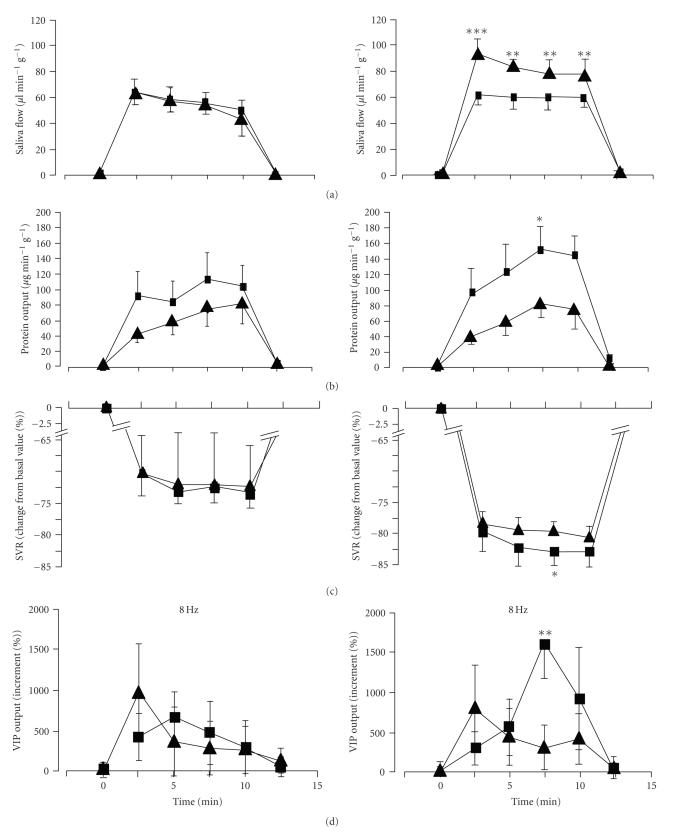
Comparison of the changes in submandibular flow of saliva (A), submandibular protein output (B), submandibular vascular resistance (C), and VIP output (D) in response to chorda tympani stimulation at 8 Hz continuously for 10 minutes (from point of time 0 to 10; indicated by horizontal bar) in the absence (▲) and in the presence (■) of methoctramine (left column of panels; 100 *μ*g kg^−1^ IV) in 5 anesthetized sheep and in the absence (▲) and in the presence (■) of pirenzepine (right column of panels; 40 *μ*g/kg iv) in 6 anesthetized sheep. Values are means ± S.E.M.

The intravenous administration of methoctramine 100 *μ*g kg^−1^ (*n* = 5) had no effect neither on the flow of saliva (Figure 1(a)), the vascular resistance (Figure 1(c)), or on the submandibular output of VIP (Figure 1(d)). The overall output of protein increased in the presence of methoctramine (+110 ± 36%; *P* < .001), although no significance was attained for the separate points in time (Figure 1(b)). Pirenzepine (40 *μ*g kg^−1^ IV; *n* = 6) significantly reduced the flow of saliva by about 30%. The overall vasodilator (+10 ± 5%; *P* < .01) as well as the protein secretory responses (+119 ± 30%; *P* < .001) both increased significantly. Both responses also attained a significant increase during the second half of the stimulation period.

### 3.3. VIP Release

The concentration of VIP in the submandibular venous effluent plasma during chorda tympani stimulation at 8 Hz rose steadily during the stimulation period. The basal release of VIP in the absence of stimulation amounted to 0.09 ± 0.03 pmol mL^−1^. When the chorda tympani nerve was challenged by the electrical stimulation in the absence of antagonist, the VIP output increased by 9 to 11 times. After the intravenous administration of methoctramine, the total mean VIP output over the 10-minutes stimulation was not significantly increased. In the presence of pirenzepine, no changes in the VIP occurred during the first two periods of stimulation, whereas it was conspicuously increased in the third period of stimulation (increased 16 times; *P* < .01; Figure 1(d)).

### 3.4. Immunohistochemistry

**Figure 2:**
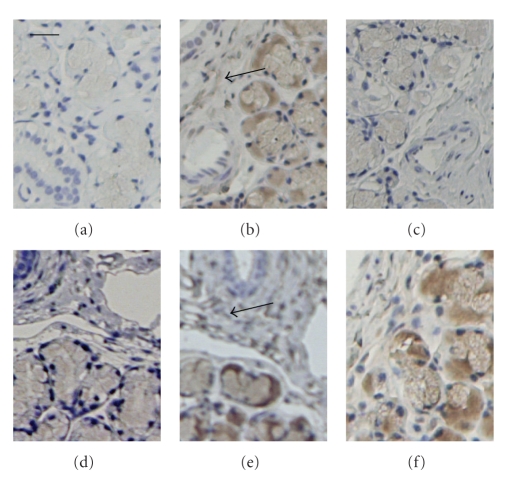
Immunohistochemical labelling of submandibular glands. Images demonstrate staining in absence of antibody (a; control); staining in the presence of muscarinic M1 (b), M2 (c), M3 (d), M4 (e), and M5 (f) receptor antibodies. Bar in panels indicates 10 *μ*m.

In the immunohistochemical examination, all muscarinic receptor subtypes except the M2 receptor were detected in the submandibular acinar tissue (Figure 2). In the glandular stroma, clear staining for the muscarinic M4 receptor appeared. Occaisonally, a vague staining for the muscarinic M1 receptor seems to occur also, but no staining was detected interacinarily for the other subtypes.

## 4. Discussion

By studying the functional responses to the stimulations, correlates to the variations in the VIP output were searched for. By immunohistochemistry examination, morphological correlates were also looked for. The functional parameters, that is, fluid and protein secretion and vasodilator responses, were almost identical to those reported previously [1]. In the current report, observations were only performed at a high frequency of stimulation (8 Hz). Neuropeptides, such as VIP, are preferentially released at intense stimulation of the nerve. Previously, activation of prejunctional muscarinic receptors has been shown to inhibit the release of VIP in salivary glands of cats, ferrets, sheep, and rats [6, 18–20], and the effect on the neuronal release has been shown to have impact on secretion as well as on vasodilatation [16, 20]. In other organs, the pharmacological characterization of muscarinic prejunctional inhibitory receptors suggests the receptors to be of either the M2 or the M4 subtype [17, 23–27]. However, morphological observations made in salivary glands indicate that prejunctional muscarinic receptors could be of the M1, M4, and the M5 receptor subtypes [28, 29]. In experiments on knockout mice, the inhibitory muscarinic receptors located prejunctionally have been shown to be of the M4 subtype and not muscarinic M2 receptors [30]. 

In the current experiments, pirenzepine reduced the nerve stimulation-induced increases in salivary flow. Even though there exists VIP-evoked as well as an atropine-resistant parasympathetic fluid response; muscarinic receptor stimulation is the principal stimulus for fluid secretion in the actual gland [1, 6]. In view of the small VIPergic response, the pirenzepine inhibition of the flow of saliva evoked by parasympathetic nerve stimulation is likely to be an effect on glandular muscarinic receptors, which is in accordance with our previous report showing the occurrence of muscarinic M1 receptors on acinar cells [1]. However, all other parameters, that is, protein output, vasodilatation, and VIP release, were increased after pirenzepine administration. Although pirenzepine preferentially binds to muscarinic M1 receptors, the selectivity window of muscarinic antagonists is very narrow and to say that the antagonists are selective for a specific subtype is erroneous, even for pirenzepine. Pirenzepine shows about 100 times greater affinity for M1 than for M2 receptors, but it shows only about 5 times greater affinity for the M1 over the M4 receptor [21]. An effect by pirenzepine on acinar excitatory receptors (e.g., M1 receptors) would of course result in a reduction of the fluid response as presently was observed. If this had been the only effect, the protein output would also have been diminished. However, since it increased in spite of the reduction of the fluid response, the reason must be found in increased stimulation of protein secretion, for instance, by a potent protein secretagogue such as the cotransmitter VIP [4]. An antagonistic effect resulting in increased responses is produced by blockade of inhibitory receptors (muscarinic M2 or M4 receptors). Concerning the affinity of the currently used antagonists on these receptors, it is close to identical on M4 receptors, whereas methoctramine has 30 times greater affinity on M2 receptors than pirenzepine. While pirenzepine significantly increased the protein output, the vasodilatation and the release of VIP, methoctramine showed tendencies towards the same pattern. Therefore, an effect via M4 receptors is more likely than an effect on muscarinic M2 receptors. 

A striking phenomenon within the current results is that the release of VIP and the VIP archetypical responses, that is, protein secretion and vasodilatation, went in parallel. The fact that the blockade caused increases in the responses favours the idea that the effects are caused by blockade of inhibitory receptors. In the morphological examination, muscarinic receptors seem to occur in stromal parts of the glandular tissue, which is in accordance to where nerve fibres histologically have been described [31]. In the current morphological examination, staining for the muscarinic M4 receptor and possibly also for the muscarinic M1 receptor was detected. Since no muscarinic M2 receptors have been detected in the morphological examinations, neither currently nor in previous observations on the ovine submandibular gland [1], it seems reasonable to conclude that inhibitory muscarinic receptors of the M4 subtype are localized prejunctionally. Since prejunctional facilitator receptors of the M1 subtype have been described in other salivary glands [17]; such receptors could possibly occur in the ovine submandibular gland also. All in all, the current observations show that inhibitory muscarinic receptors modulate the neuronal release of transmitters in the submandibular gland of the sheep and that these are likely to be of the muscarinic M4 receptor subtype.
